# Opposite development of short- and long-range anterior cingulate pathways in autism

**DOI:** 10.1007/s00401-018-1904-1

**Published:** 2018-09-06

**Authors:** Basilis Zikopoulos, Xuefeng Liu, Justin Tepe, Iris Trutzer, Yohan J. John, Helen Barbas

**Affiliations:** 1 Human Systems Neuroscience Laboratory, Boston University, 635 Commonwealth Ave., Room 401D, Boston, MA 02215 USA; 2 Program in Neuroscience, Boston University, Boston, MA 02215 USA; 3 Neural Systems Laboratory, Boston University, Boston, MA 02215 USA

**Keywords:** White matter, Axon, Prefrontal cortex, Myelin, Fractional anisotropy

## Abstract

Autism has been linked with the changes in brain connectivity that disrupt neural communication, especially involving frontal networks. Pathological changes in white matter are evident in adults with autism, particularly affecting axons below the anterior cingulate cortices (ACC). It is still unknown whether axon pathology appears early or late in development and whether it changes or not from childhood through adulthood. To address these questions, we examined typical and pathological development of about 1 million axons in post-mortem brains of children, adolescents, and adults with and without autism (ages 3–67 years). We used high-resolution microscopy to systematically sample and study quantitatively the fine structure of myelinated axons in the white matter below ACC. We provide novel evidence of changes in the density, size and trajectories of ACC axons in typical postnatal development from childhood through adulthood. Against the normal profile of axon development, our data revealed lower density of myelinated axons that connect ACC with neighboring cortices in children with autism. In the course of development the proportion of thin axons, which form short-range pathways, increased significantly in individuals with autism, but remained flat in controls. In contrast, the relative proportion of thick axons, which form long-range pathways, increased from childhood to adulthood in the control group, but decreased in autism. Our findings provide a timeline for profound changes in axon density and thickness below ACC that affect axon physiology in a direction suggesting bias in short over distant neural communication in autism. Importantly, measures of axon density, myelination, and orientation provide white matter anisotropy/diffusivity estimates at the level of single axons. The structural template established can be used to compare with measures obtained from imaging in living subjects, and guide analysis of functional and structural imaging data from humans for comparison with pathological states.

**Electronic supplementary material:**

The online version of this article (10.1007/s00401-018-1904-1) contains supplementary material, which is available to authorized users.

## Introduction

Autism spectrum disorders (ASD) have been linked with the changes in brain connectivity that disrupt neural communication [4, 5, 23, 79]. Frontal networks, in particular, show gross changes, with local over-connectivity, and long-range under-connectivity in ASD [1, 5, 19, 20, 37–39, 46, 66, 67]. We previously found that the white matter below the anterior cingulate cortex (ACC) in the brains of adults with ASD has more thin axons that connect ACC with the nearby areas and fewer thick axons that extend over long distances [79, 80, 84]. These findings revealed pathology in the fine structure of axons in adults with ASD, but it is unknown whether the changes appear early and persist or change in the course of development.

Moreover, both typical and atypical development of cortical pathways remain grossly understudied at the single axon level. In view of the appearance of ASD symptomatology at an early age, we reasoned that the disorganization of axons below the ACC may start early. Comparison with adults may thus provide insight about the developmental trajectory for both typical and disrupted neural communication.

To begin to address these issues, we used post-mortem brain tissue from children (ages 3–10 years), teenagers (ages 14–15 years) and adults (ages 30–67 years) with and without ASD, and systematically sampled and imaged individual myelinated axons in the white matter below ACC at high resolution in the light (LM) and electron microscope (EM). Our goal was to investigate the typical postnatal development of vulnerable ACC pathways [29] and compare with the developmental trajectory of axons in ASD.

We provide evidence for systematic changes in the density, size and trajectories of axons of ACC pathways in typical postnatal development from childhood through late adulthood. Against the normal profile of development, the ASD groups showed lower density of myelinated axons in childhood, followed by an increase in adulthood in pathways that travel over short distances and a decrease in pathways that course over long distances, in patterns that differed significantly from the control groups. These findings reveal fine structural underpinnings of persistent pathology that may be key anatomical correlates of disrupted neural communication in ASD.

## Methods

### Experimental design

In this investigation of ACC white matter development, we focused on myelinated axons, because neural pathways are known from classic studies to gradually myelinate from childhood to adulthood [48, 77]. The goal was to study the fine structure of axons that affect neural communication, including their density, size, orientation, and relationship to myelin in two parts of the white matter: the superficial white matter (SWM), which carries axons that mostly course over short or medium distances, and the deep white matter (DWM), which carries axons that course over long distances [32, 33, 62]. In this study, we sampled an extensive paracingulate (anterior to the corpus callosum) and cingulate region of white matter at the optical and electron microscope, covering approximately 1.5 cm^3^ beneath cortical areas 32 and anterior 24, collectively called ACC here. At levels of the cingulate that surrounded the genu of the corpus callosum, we included both dorsal (above the corpus callosum) and ventral (subgenual) ACC regions, as described in detailed cytoarchitectonic studies and atlases mapping the human brain [42, 43, 49, 50, 56, 69, 70, 72, 73].

### Human post-mortem brain acquisition and tissue preparation

We obtained age- and gender-matched post-mortem brain tissue that was immersion fixed in 10% formalin, from 32 individuals, 15 neurotypical controls (CTR) and 17 with ASD (Table 1 and Supplementary Table 1). Brain tissue was obtained from the Autism Tissue Program, the Harvard Brain Tissue Resource Center, the Institute for Basic Research in Developmental Disabilities, the University of Maryland Brain and Tissue Bank, the National Disease Research Interchange (NDRI), and Anatomy Gifts Registry. Clinical characteristics, including autism diagnostic interview scores, and other data are summarized in Table 1 and Supplementary Table 1. Cases were matched as closely as possible based on tissue availability and the study was approved by the Institutional Review Board of Boston University. Despite limited tissue availability, we obtained well-preserved and appropriately stored tissue with a short PMI (on average < 24 h, and < 16 h in about half of the cases). Our sample included one ASD case with a PMI of 99 h; however, the mean PMI overall was relatively low and comparable to the mean PMI of cases used in studies that have reported similar estimates. Signs of post-mortem autolysis were minimal in myelinated axons and the white matter, and were restricted to minor dissociation of the myelin sheath or small inclusions in the axolemma in some thick axons, and the typical ‘fried egg’ appearance of oligodendrocytes in post-mortem tissue, with a seemingly intact nucleus positioned in a bloated, but empty cytoplasm. In the gray matter, broken membranes from cell processes were also apparent. We have developed a series of methods to preserve tissue quality even after extensive processing and multiple labeling, as elaborated below. These include storing tissue blocks or sections in anti-freeze solution at − 20 °C and using a variable wattage microwave oven to improve penetration of agents and optimize specificity of labeling [27, 28, 80, 84–86].

**Table 1:** Clinical characteristics of patients with autism and data on neurotypical individuals in the study

Subject number	Group	Sex	Age (year)	PMI (h)	Primary cause of death
451	CTR	M	4.6	15	Accidental drowning
4337	CTR	M	8.2	16	Blunt force neck injury
3835	CTR	F	9.6	8	Asphyxia
356	CTR	M	8.1	14	Accident, injuries
1548	CTR	M	10		Unknown
1670	CTR	M	13.3	5	Respiratory distress
4722	CTR	M	14.5	16	Multiple traumatic injuries
1706	CTR	F	8.6	20	High fever—rejection of cardiac transplant
4203	CTR	M	7.8	24	Respiratory insufficiency
HAW	CTR	F	58	30	Pancreatic cancer
HAY	CTR	M	67	30	Pancreatic cancer
B-4786	CTR	M	36	20	Myocardial infarction
B-4981	CTR	M	42	18	Myocardial infarction
B-5353	CTR	F	41	14	Unknown
B-6004	CTR	F	36	18	Unknown
Mean CTR			24.3	17.7	
5144	ASD	M	7.2	3	Cancer
4021	ASD	M	3.3	15	Accidental drowning
4029	ASD	M	3.8	13	Accidental drowning
5308	ASD	M	4.5	21	Accident, injuries
1182	ASD	F	10	24	Smoke inhalation
AN01293 (B-6349)	ASD	M	9	4	Cardiopulmonary arrest
AN03221	ASD	M	7	11	Accidental drowning
AN03345 (B-6399)	ASD	M	2.8	4	Cardiac arrest
AN13872 (B-7002)	ASD	F	5.6	33	Accidental drowning
AN08873 (B-5569)	ASD	M	5	25.5	Accidental drowning
AN04682 (B-7079)	ASD	M	15	23.2	Asphyxia, hanging
HSB4640	ASD	M	8.5	14	Asthma attack, seizure, high fever
AN-06746 (B-4541)	ASD	M	44	31	Acute myocardial infarction
AN-18892 (B-4871)	ASD	M	31	99	Shooting
AN-08792 (B-5173)	ASD	M	30	20	Gastro-intestinal bleeding
AN-07770 (B-6232)	ASD	F	40	33	Respiratory arrest
AN-11989 (B-6677)	ASD	M	30	16	Congestive heart failure
Mean ASD			15.1	23.2	

*PMI* post-mortem interval

The diagnosis of autism was based on the Autism Diagnostic Interview-Revised (ADI-R, Table S1). One child and an adult with ASD were diagnosed with seizure disorder (HSB4640, AN 08792), and two other adults were diagnosed with depression (AN 18892), and schizophrenia (AN 06746). Results from the analysis of the features of axons in these subjects as well as the female subjects did not differ from others within each group, in this and other studies that used tissue from the same cases [9, 78, 80, 84]. We further scrutinized the brain from a 67-year-old adult in the CTR group (case HAY), to determine whether there were normal aging-related changes in myelin. Macroscopic and histological examination of the brain tissue from this case at the light and electron microscopic level did not reveal any remarkable or unusual findings. Further quantification of gray and white matter features of this case has produced estimates well within the range of estimates from other CTR cases, consistent with previous [27, 28, 84], and ongoing studies. In line with our assessment, changes in myelin after the age of 60 years have not been reported for the ACC, which is one of the least myelinated regions of the cortex [84]. Detailed histological studies at the light and electron microscopic level by Peters et al. have shown modest changes in late adulthood in the myelin sheath of a few axons in lateral prefrontal and striate visual cortices of non-human primates, but no changes in the density of myelinated axons [47, 53, 55]. Imaging (MRI) studies in humans have reported changes in the levels of white matter myelin in late adulthood in lateral frontal, medial temporal and temporoparietal cortices, but not in ACC or primary sensory and motor cortices [8, 76]. Some of the changes detected with MRI, using T2 and DTI imaging methods may reflect changes in the permeability of the blood–brain barrier that lead to changes in interstitial water content that can be mistaken for changes in myelin [31]. We therefore, included data from this case for analysis.

**Fig. 1:**
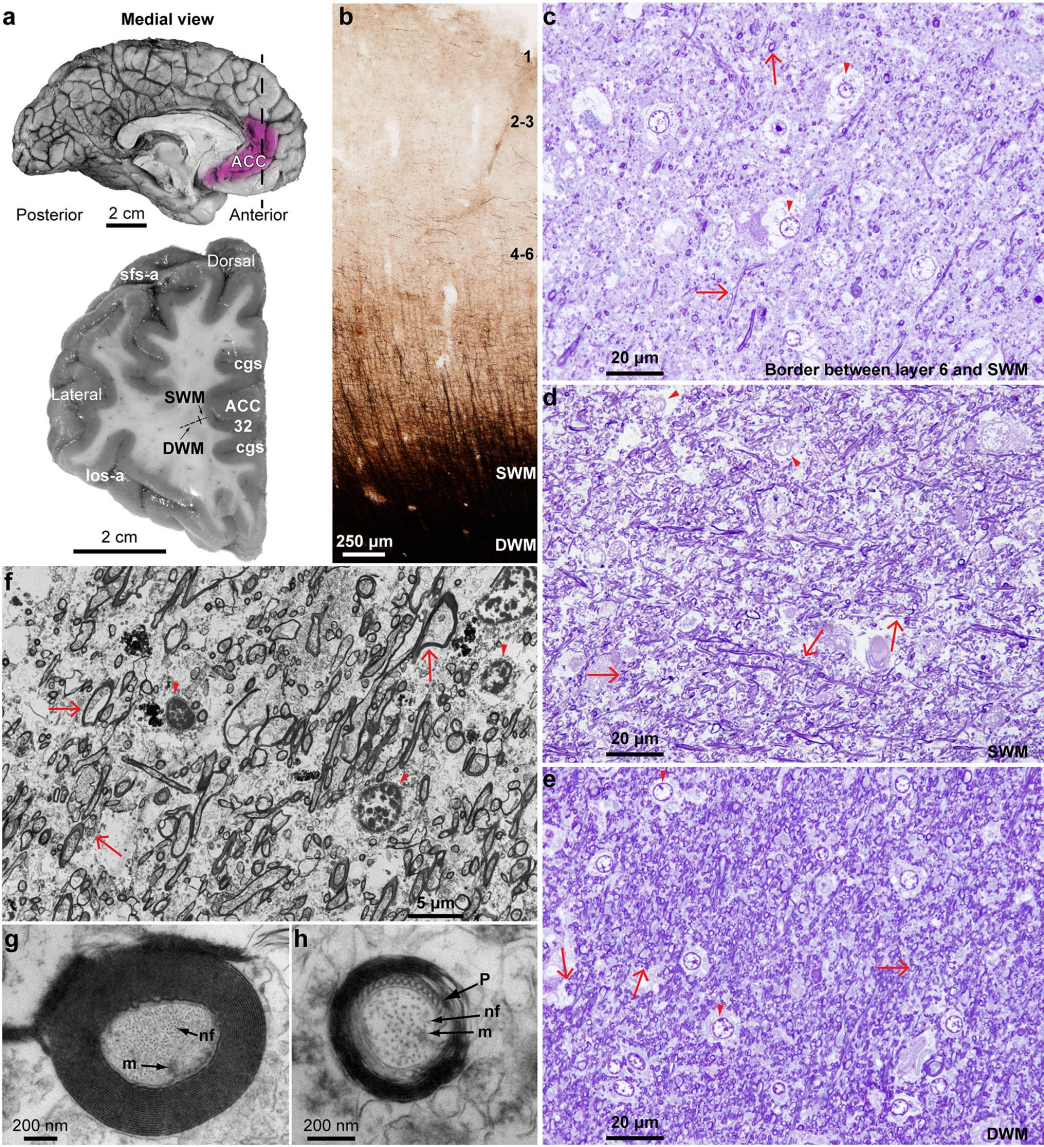
Experimental design. **a** Medial view of the left hemisphere of the human brain (top) and a frontal coronal tissue slab (below) that was taken at the level marked by the dotted line (top). The section shows the anterior cingulate region in the medial prefrontal cortex (ACC) at the level of Brodmann’s area 32. The tissue slab shows approximate segmentation of the white matter into SWM and DWM regions. **b** Myelinated axons in a representative cortical column of area 32 (gray matter layers 1–6 and SWM, DWM) from a 50-μm-thick coronal section stained with the Gallyas silver method (case HAW). The ACC exhibits low to moderate levels of myelination in the human brain. **c**–**e** Photomicrographs of 1-μm-thick coronal sections from ACC through the bottom of layer 6 (**c**, case B-6004), SWM (**d**, case B-4786), and DWM (**e**, case B-6004) stained with toluidine blue. Red arrows point to examples of myelinated axons and red arrowheads point to oligodendrocytes. Note the increase in the density of myelinated axons deep in the cortical column and white matter. **f**–**h** Electron photomicrographs of ultrathin (50 nm) coronal sections that show the ultrastructure of the white matter below ACC at high resolution (EM). Myelinated axon profiles can be identified by the darkly stained electron-dense myelin (red arrows in f, case HAW). The quality of the tissue was good enough (e.g.,** g**, case 3835 and h, case 4337) to identify microtubules (m), neurofilaments (nf), the spiraled lamellae of the myelin sheath that surround the axolemma, and pockets of paranodal cytoplasm (P) that are found in the vicinity of unmyelinated nodes, where axons branch, and were only seen in the SWM near the border with layer 6

We used coronal ACC blocks from formalin-fixed post-mortem brain tissue (Fig. 1a), matched based on the human brain atlas [43] and [73], and additional cytoarchitectonic studies of human prefrontal cortex [60, 72]. We postfixed tissue slabs in 2% paraformaldehyde and 2.5% glutaraldehyde, in 0.1 M phosphate buffer (PB, pH: 7.4) for 2–4 days at 4 °C. To preserve the ultrastructure until processing, we cryoprotected tissue blocks in 25% sucrose solution and then immersed them in anti-freeze solution (30% ethylene glycol, 30% glycerol, 40% 0.05 M PB, pH: 7.4 with 0.05% azide) before storing at − 20 °C [80, 84]. Tissue blocks were then rinsed in 0.1 M PB and cut coronally at 50-μm-thick sections on a vibratome (Pelco, series 1000). Some blocks were frozen in − 70 °C isopentane and cut in a cryostat (CM 1500, Leica), or a sliding microtome (AO), equipped with a PhysiTemp freezing platform, in the coronal plane at 20–50 µm in 10 series of free floating sections. Sections used for Nissl histological stains were mounted on chrome-alum gelatin-coated slides.

### Nissl and Gallyas staining of thick sections for optical microscopy

Using standard histological methods, we stained series of sections with Nissl (thionin) or the Gallyas silver myelin stain to visualize neurons, glia, and intracortical myelin with the optical microscope (Fig. 1) [28, 86]. Briefly, we mounted series of 20–50-μm-thick sections of human ACC on gelatin-coated slides (Gelatin Type A, G8-500, Fisher Scientific, Fair Lawn, NJ, USA) and after drying for a week, we stained them for Nissl using thionin blue (Thionin powder, T-409, Fisher Chemicals) to view neurons and glia and examine the cortical cytoarchitecture, as described [28, 84]. Briefly, we defatted sections in a 1:1 solution of chloroform (C298-1, Fisher Scientific) and 100% ethanol (Pharmco-AAPER, Brookfield, CT, USA) for 1–3 h, before rehydrating through a series of graded alcohols and dH_2_O, and then staining with 0.05% thionin (pH 4.5) for 15 min. Sections were differentiated through graded alcohols, cleared with xylenes (UN1307, Fischer Scientific) and coverslipped with Entellan (Merck, Whitehouse, NJ).

We stained other series of sections using the Gallyas silver technique to label intracortical and white matter myelin [26, 48, 86]. Briefly, sections were rinsed in distilled water and then incubated in a solution of pyridine (2/3; P368-1 Fischer Scientific) and glacial acetic acid (1/3; ARK2183 Sigma-Aldrich) for 30 min at room temperature. We then washed sections in distilled water before incubation in the impregnation solution for a minimum of 30 min at room temperature in the dark. We used 0.1 g of ammonium nitrate (A7455 Sigma-Aldrich) and 0.1 g of silver nitrate (S181-25 Fischer Scientific) per 100 ml of distilled water to make the impregnation solution; the pH of the solution was adjusted with 0.1 M sodium hydroxide (CAS 1: 1310-73-2 Fischer Scientific) to obtain pH 7.5. Sections were then rinsed in 0.5% acetic acid (A6283 Sigma-Aldrich) and incubated in the developing solution under microscopic control until the proper level of stain was achieved. For the developing solution we first prepared three solutions, each dissolved in 500 ml of distilled water: A, 25 g sodium carbonate (S-263 Fischer Scientific); B, 1 g ammonium nitrate (A7455 Sigma-Aldrich), 1 g silver nitrate (S181-25 Fischer Scientific) and 5 g silicotungstic acid (383341 Sigma-Aldrich); C, 75 ml of solution B and 1.75 ml of 4% paraformaldehyde (O4042 Fischer Scientific). To make the incubation solution, we mixed 150 ml of solution A, 75 ml of solution B and 75 ml of solution C in that order. After developing, the sections were washed in 1% acetic acid (A6283 Sigma-Aldrich) and then in distilled water followed by incubation in 5% sodium thiosulfate (S-1648 Sigma-Aldrich) to stabilize the reaction. Sections were finally washed in distilled water and mounted on gelatin-coated glass slides from PB 0.1 M, pH 7.4.

### Electron microscopy processing, cutting, and staining

We processed adjacent series of 50-μm-thick coronal sections for EM, using a high-contrast method. We confirmed the presence of ACC cortical gray matter in adjacent sections that were stained with Nissl (see above). We rinsed sections in 0.1 M PB and postfixed them in a variable wattage microwave oven (Biowave, Pelco) with 6% glutaraldehyde at 100–150 W. Under a dissecting microscope, we cut small regions of sections containing the superficial or deep parts of the white matter below ACC. White matter regions of interest were rinsed in 0.1 M cacodylate buffer, and then postfixed with intermediate water rinses first in 0.1% tannic acid, then in 1% osmium tetroxide with 1.5% potassium ferrocyanide, then in TCH aqueous solution (0.1 g of thiocarbohydrazide), and finally in 2% osmium tetroxide. Sections were washed in water, stained overnight with 1% uranyl acetate followed by lead aspartate and dehydrated in an ascending series of alcohols. Tissue sections were then cleared in propylene oxide and embedded in araldite or LX112 resin at 60 °C and then in Aclar film for storage. Pieces of the resin-embedded sections were cut and re-embedded in resin blocks. We cut serial semithin (1 μm) or ultrathin (50-100 nm) coronal sections with a diamond knife (Diatome, Fort Washington, PA), using an ultramicrotome (Ultracut; Leica, Wein, Austria). Lipids in the myelin sheath and in membranes were rendered electron-dense and appeared dark after EM processing [54].

For the Nissl staining of semithin sections, we prepared 1% aqueous solution with toluidine blue powder (T3260, Sigma-Aldrich) in distilled water. The solution was filtered and mixed with sodium borate (1:1; S9640, Sigma-Aldrich), filtered again and diluted 1:1 with 70% ethanol. Semithin sections floating in water were mounted on gelatin-coated slides and placed on a heater plate until the water evaporated. Sections were then covered with the final toluidine blue solution for 1 min, rinsed with water, differentiated with 70% ethanol and rinsed with water, before coverslipping as described above.

We collected ultrathin sections on single slot pioloform-coated grids to view with a transmission electron microscope (100CX; Jeol, Peabody, MA, USA) or a scanning electron microscope (Zeiss Gemini 300 with STEM detector and Atlas 5 software modules), at magnifications between 2000 and 50,000×, as described [28, 80, 84].

### Unbiased estimate of neurons, glia, and myelinated axons

We estimated the overall density of neurons, astrocytes, and oligodendrocytes in ACC gray matter regions of interest (ROIs) overlying the white matter regions examined, based on previous maps of the human brain [43, 73]. For each case, we traced a minimum of three gray matter ACC ROIs of equal width from one series of coronal sections that were on average 500 μm apart. We then used the optical fractionator method [30, 34] to sample the outlined ROI volume using an unbiased systematic random procedure, with the aid of commercial software (StereoInvestigator; Microbrightfield, Williston, VT, USA), as described [29, 80, 86].

We counted Nissl-stained neurons, astrocytes, and oligodendrocytes in the outlined ROI volume at 1000×, using systematic random sampling. We identified neurons and glia based on their characteristic features, following a detailed cytology algorithm we have developed [28]. Briefly, we split labeled cells into two broad groups: the first group included small cells with darkly stained nuclei (microglia and oligodendrocytes) and the second group included larger cells with lighter nuclear stain (neurons, astrocytes, and endothelial cells). We then followed the detailed neurocytology algorithm [28] to distinguish among different cell types, using key cytological features. These included the presence or absence of cytoplasm around the nucleus (present in neurons; absent in glia and endothelial cells), the distribution of heterochromatin, and the staining of euchromatin in the nucleus. Rounded and darkly stained nuclei with 2–4 granules of heterochromatin, often with perinuclear halo and/or a pinkish crescent of cytoplasm, were typical of oligodendrocytes. Cells with lightly stained nuclei and unstained cytoplasm, with a rim of peripheral heterochromatin under the nuclear envelope and several heterochromatin granules attached to this rim or in the heterochromatin net, were classified as astrocytes. The features of neurons included lightly stained nuclei, often with visible folding of the nuclear membrane, and stained cytoplasm.

We set the counting frame (disector) size for cell counts at 50–60 μm with a height of 5 μm and grid spacing of 100–300 μm. To ensure unbiased estimate of the number of cells we first measured the thickness of each section, and set a guard zone at the bottom and top of each section to correct for objects plucked during sectioning (minimum 2 μm in 10–15 μm sections after tissue shrinkage); the disector thickness was thus smaller than the thickness of the section [30, 34, 75]. These parameters yielded a sampling fraction with a coefficient of error < 10% per contour, as recommended [30, 34]. The use of uniform random sampling ensured that every part of each region of interest examined had the same chance of being included in the sample. We computed cell density by dividing the estimated number of counted cells within each ROI with the estimated volume of each ROI (from the planimetry estimates) to assess the relative density (cells/volume of ROI in mm^3^).

Following a similar approach, we used uniform random sampling to image axons systematically at the optical microscope. To estimate axon density, we captured images of semithin toluidine blue-stained white matter sections at 1000× with the same light exposure to minimize background variability. We then converted to grayscale, inverted, and thresholded images in ImageJ so that myelinated axons were highlighted, and we estimated the ratio of the surface area of myelinated axons within each sample and averaged for each subject.

We estimated the relative density of axons and the thickness of axons and myelin in the white matter below ACC at the EM, using a systematic random sampling fraction of 1:1,000 that yielded more than 6000 axons, per case, per ROI. We divided the white matter into a superficial part (close to the gray matter, SWM) and a deep part (DWM), as described (Fig. 1) [80, 84]. We captured high-resolution images of areas of interest that were imported in ImageJ or Reconstruct [24] and calibrated. To estimate the overall density of axons, we divided the surface area of axon profiles by the total surface area of the sampled region, imaged at low magnification (2000–3300×). We estimated the inner and outer diameter as well as the thickness of the surrounding myelin sheath at high magnification (10,000–50,000×), as described [80]. In our analysis, we included all axon profiles: those that were perpendicular to the cutting plane and appeared circular, as well as elongated profiles. To ensure consistency, we measured the inner and outer diameters perpendicular to the center of the maximum diameter of the axon profile.

### White matter segmentation and axon orientation analysis

We subdivided white matter beneath ACC in two regions, SWM and DWM, by determining axon alignment in semithin toluidine blue-stained sections under the microscope, at gradually increasing distances from the gray–white matter border, as described (Fig. 1, [80, 84]). The SWM was immediately adjacent to layer 6 of the overlying ACC, and included axons that run mainly radially to the surface of the cortex, and was about 1.5 mm in depth [36, 62, 79, 80]. The border between layer 6 and SWM in frontal limbic cortices of the primate brain is irregular, characterized by an indistinct transition zone ([21, 22], and personal observations), which is exaggerated in ASD [6, 64]. To take into account the irregular cell patterning at the cortical gray–white matter boundary, we included the bottom of layer 6 in the SWM sampled region. The DWM included axons that run mainly sagittally to the cerebral surface [36, 62, 79, 80].

**Fig. 2:**
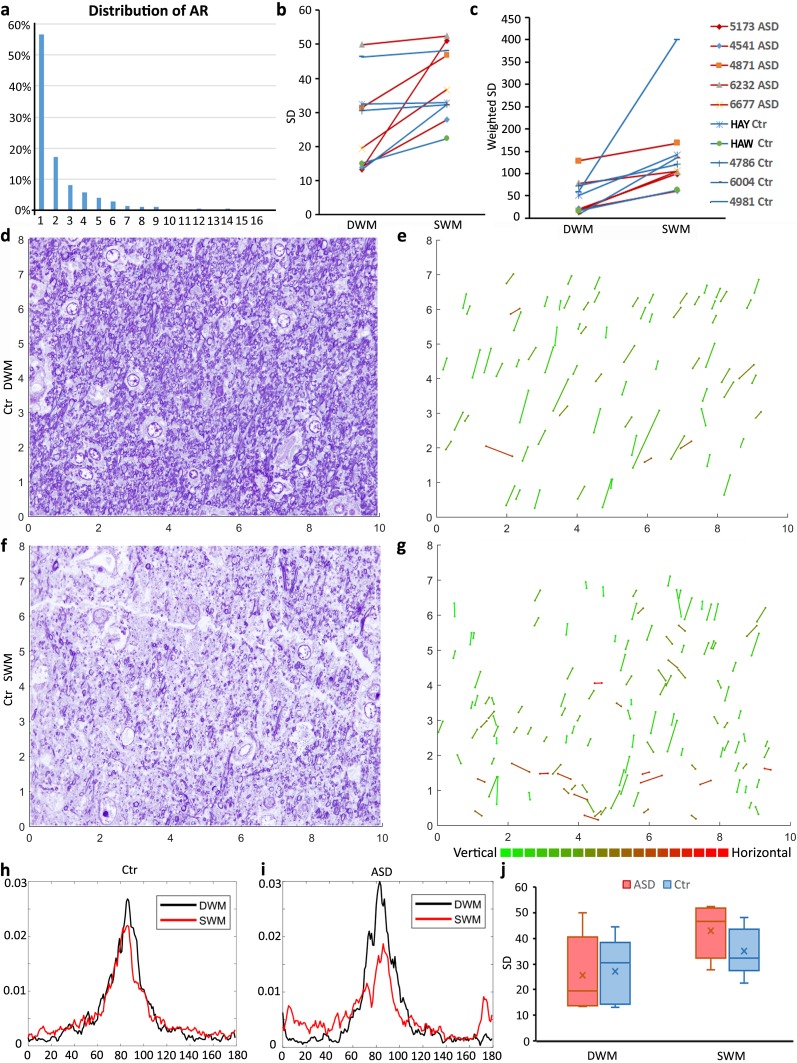
Myelinated axon trajectory below ACC. **a** Representative histogram of the cross-sectional aspect ratio (AR) of all myelinated axon profiles traced in 1-μm-thick sections stained with toluidine blue. Smaller AR indicates that the axon is more perpendicular to the image plane, increasing the likelihood of inaccurate orientation estimation in 2D images. Therefore, we performed axon trajectory analysis using axon segments with AR ≥ 3 (approximately 30% of all axons), or, for a comprehensive assessment of all axons, normalizing based on the proportion of elongated axon profiles (AR ≥ 3) within the entire complement of axons in each case. Both approaches yielded similar results. **b**, **c** Significantly higher axon orientation variability in SWM, represented by larger standard deviation (SD) of axon angle values in SWM compared to DWM in each case, with or without normalization [paired *t* test to compare SD values between SWM and DWM; *N* = 10 (CTR and ASD cases pooled); **b** (SD), two-tailed *p* value = 0.011974, one-tailed *p* value = 0.005987; **c** (weighted SD, see methods), two-tailed *p* value = 0.01514, one-tailed *p* value = 0.00757]. **d**–**g** Representative images and plots of toluidine blue-stained ACC sections from a control (neurotypical) brain, at DWM (**d**, case B-6004) and SWM (**f**, case B-6004). **e**, **g** Stick plots highlighting the distribution of elongated axon profiles, traced from the corresponding original images in** d** and** f**, respectively. Sticks are color-coded to represent specific orientations. Axons plotted in DWM are of similar color (**e**), indicating more homogeneous orientation distribution. Axons in SWM have more heterogeneous orientation distribution (**g**). **h**, **i** Average axon trajectory angles from all images analyzed in the CTR (**h**) and ASD (**i**) groups for SWM and DWM. In both groups, the distribution for DWM axons appears sharper than for SWM, indicating smaller variability. **j** Summarized box-plot graph shows increased heterogeneity of axon trajectory in SWM compared to DWM, and also revealed a trend for modestly higher axon orientation variability in the ASD compared to the CTR group in SWM below ACC

We used uniform random sampling of semithin toluidine blue-stained sections to capture images in the SWM and DWM beneath ACC with the optical microscope at 1,000x magnification. We imported images in ImageJ to outline profiles of myelinated axons, and estimate morphometric parameters including major and minor diameters that were used to estimate the axon profile aspect ratio (circularity), and orientation angle. We estimated myelinated axon orientation variability within each white matter region by measuring axon profile angle trajectories in each image and then calculating the standard deviation (SD) in each image (Fig. 2b, c). We analyzed 2–3 images, each with about 2000 axon profiles, per case, and SD values for each case and region of interest were averaged for further statistical comparison. Based on previous analysis of 3D serial EM images in the human and rhesus monkey white matter [80, 81, 83], we constrained axon orientation analysis to elongated profiles with aspect ratio (AR = major diameter/minor diameter) ≥ 3, which included, on average, about 30% of all myelinated axons (Fig. 2a). The remaining axon profiles with AR < 3 appeared more circular, because axons were mostly perpendicular to the surface of each image, resulting in estimated angle values that are not reliable indicators of axon orientation. To minimize bias on orientation difference from image acquisition, we normalized angle values of each image by aligning the peak angle to 90°. In addition, to account for all myelinated axons in our sample, we estimated the ratio of elongated/all myelinated axon profiles (circular + elongated) for each image and assigned these as weighting factors to produce weighted SD values (Fig. 2c).

### Statistical analysis and sample size

We gathered data blind to condition and white matter region and conducted independent analyses by at least two investigators. Random codes for human subjects and images were broken after completion of each part of the study. To estimate the sample size, we took into account the number of cases and volume fraction of ACC sampled so that the number of individual cells and axons examined produced estimates with a small coefficient of error (< 10%), as described [29, 80, 82, 86]. We additionally used data from our previous studies of cell and axon densities, prior pilot studies with exhaustive sampling, progressive means analysis, and the formula of West et al. [75], taking into consideration all known and estimated variables, including age, sex, post-mortem interval (PMI), and other diagnoses. Combined, these analyses showed that the sampling ratios used exceeded the samples needed to detect differences with a greater than 90% probability and with an estimated large effect size in the population (0.80).

It should be noted that, to the best of our knowledge, this study included the largest sample size and cohort of brain tissue from post-mortem human cases examined to date at such high resolution. We imaged and analyzed an estimated 1,000,000 individual myelinated axons from post-mortem brain tissue from 32 individuals. Most axons were imaged at 1000× magnification with the optical microscope, where we could examine large white matter regions of interest and reliably resolve myelinated axons down to 0.2 μm in diameter. A quarter of the axons were independently imaged at high resolution in the EM (2000–50,000× magnification), where we could reliably identify all myelinated axons, including about 20% with diameters below 0.2 μm thickness. For the EM analysis of axon densities and sizes, we obtained samples from widely spaced ultrathin sections (one every ten) and fields of view through systematic random sampling to minimize the likelihood of sampling axons from the same parent branch. This sampling scheme and the fact that most axons branch very close to or after they enter the gray matter minimized the likelihood of counting segments of the same axon more than once.

We quantitatively estimated 16 morphometric structural features of myelinated axons in the white matter (feature dimensions), including: relative axon density (all myelinated axons), relative axon density (only elongated profiles with AR ≥ 3), outer diameter, inner diameter, major diameter of outlined axon profile, axon orientation, myelin thickness, g-ratio, relative proportion of thin, medium, thick and extra-large axons, myelin thickness of thin axons, myelin thickness of medium axons, myelin thickness of thick axons, and myelin thickness of extra-large axons.

We performed statistical analyses using Statistica (StatSoft, Tulsa, OK; RRID: SCR_014213), SPSS (IBM), or Matlab (MathWorks). We evaluated data through scatter and frequency distribution plots and K-means cluster analysis, with parameters set to maximize initial between-cluster distances, to segregate axons into four groups by thickness, based on their outer diameters, which included the myelin sheath. Data distributions for continuous variables were not significantly different from normal as determined by the Kolmogorov–Smirnov test, and thus allowed the use of parametric statistics. We initially used Chi square and Kolmogorov–Smirnov tests to examine axon size distributions and multiple linear regression analysis to examine correlations. To compare axon or cell densities across cases and conditions we employed one-way ANOVA or two-tailed *t* tests. We used ANCOVA, with age as a covariate, to test for differences in the developmental trajectories of axon features. For all analyses, *p* values < 0.05 were taken as statistically significant.

Finally, we examined potential effects of sex, PMI, and other diagnoses (i.e., seizures) on all estimates for axon size as well as axon and cell density, using correlation analysis. In addition, we compared all estimated variables across cases using MANCOVA with sex, PMI, and other diagnoses as covariates. These analyses did not yield additional significant effects.

### Photography

Digital images that were used for analyses were not modified. We assembled figure panels by importing images into Adobe Illustrator software (Adobe Systems Inc., San José, CA, USA). Images were not retouched or edited other than applying minor adjustments of overall brightness and contrast using Adobe Photoshop.

## Results

### Normative features of myelinated axons below ACC

We first assessed the density of myelinated axons. This analysis showed that myelinated axons made up approximately 34% of the white matter below ACC in both ASD and CTR groups. The density of myelinated axons (% of space occupied by myelinated axons) increased progressively with increasing distance from cortical layer 6 and depth in the white matter [(CTR mean ± SD: 34 ± 6% in SWM; 39 ± 13% in DWM) and (ASD mean ± SD: 28 ± 8% in SWM; 35 ± 10% in DWM)]. The rest of the white matter was occupied mostly by unmyelinated axons and glia, mainly oligodendrocytes.

We then measured axon diameters, which ranged between 0.1 and 9.7 μm, consistent with findings in human cortex [10, 41, 79, 80, 84]. The g-ratio (inner/outer diameter), an indicator of efficiency of conduction velocity and neurotransmission, increased significantly with axon size, with an average value near the optimal level of 0.6 [59]. Axons were grouped through cluster analysis based on outer diameters into thin (0.1–0.83 μm), medium (0.84–1.51 μm), thick (1.52–2.65 μm), and extra-large (thicker than 2.65 μm). In all cases, most axons were thin/medium in thickness (~ 70%). The average thickness of axons in the SWM (mean ± SD: 0.77 ± 0.09 μm) was lower than in the DWM (0.93 ± 0.14 μm) of CTR and ASD groups (SWM: 0.73 ± 0.09 μm; DWM: 0.90 ± 0.32 μm).

We next measured the orientation of myelinated axons. Results showed significantly more axon orientation variability in the SWM than in the DWM in each group (ASD and CTR) and for each individual brain (*t* test, *p* = 0.00001; Fig. 2). The SWM had fewer and overall thinner axons, which were oriented in variable directions, indicative of increased axon branching as they approach their termination site (see also Fig. 1h). By contrast, axons in the DWM traveled mostly in parallel (Fig. 2b–f). The distribution of axon trajectories did not differ significantly between CTR and ASD groups, but there was a trend for wider spread and a more abrupt change between SWM and DWM in the ASD group (Fig. 2b, h–j).

### Axon development from childhood to adulthood

**Fig. 3:**
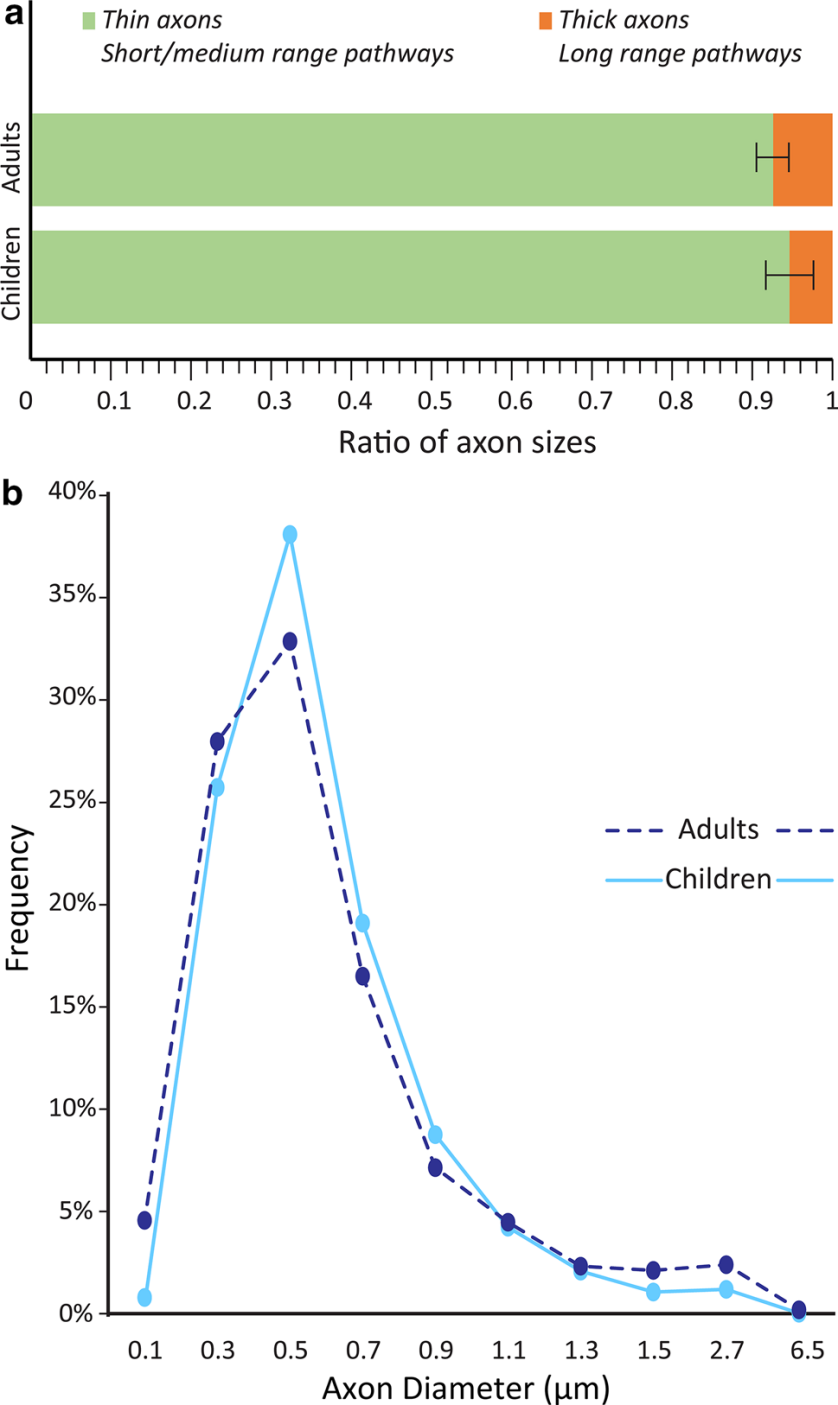
Normative postnatal development of myelinated axons below ACC. The proportion of thick axons increases with age so that adults tend to have relatively more thick myelinated axons than children. **a** Proportion of thin to thick myelinated axons in the white matter below ACC, averaged across CTR children (3–10 years, bottom bar graph) and CTR adults (30–67 years, top bar graph). Most axons in the white matter below ACC in children and adults were thin, which have been shown to take part in short-/medium-range pathways. However, adults showed a clear trend for increase in the relative proportion of thick axons that participate in long-distance pathways compared to children. Error bars indicate standard deviation. **b** Neurotypical frequency distribution of myelinated axon diameters in the white matter below ACC, averaged across all CTR children (3–10 years, light blue line) and CTR adults (30–67 years, dotted dark blue line). The distribution peak and right extreme is skewed towards fewer thin and more thick axons in adults than in children

**Fig. 4:**
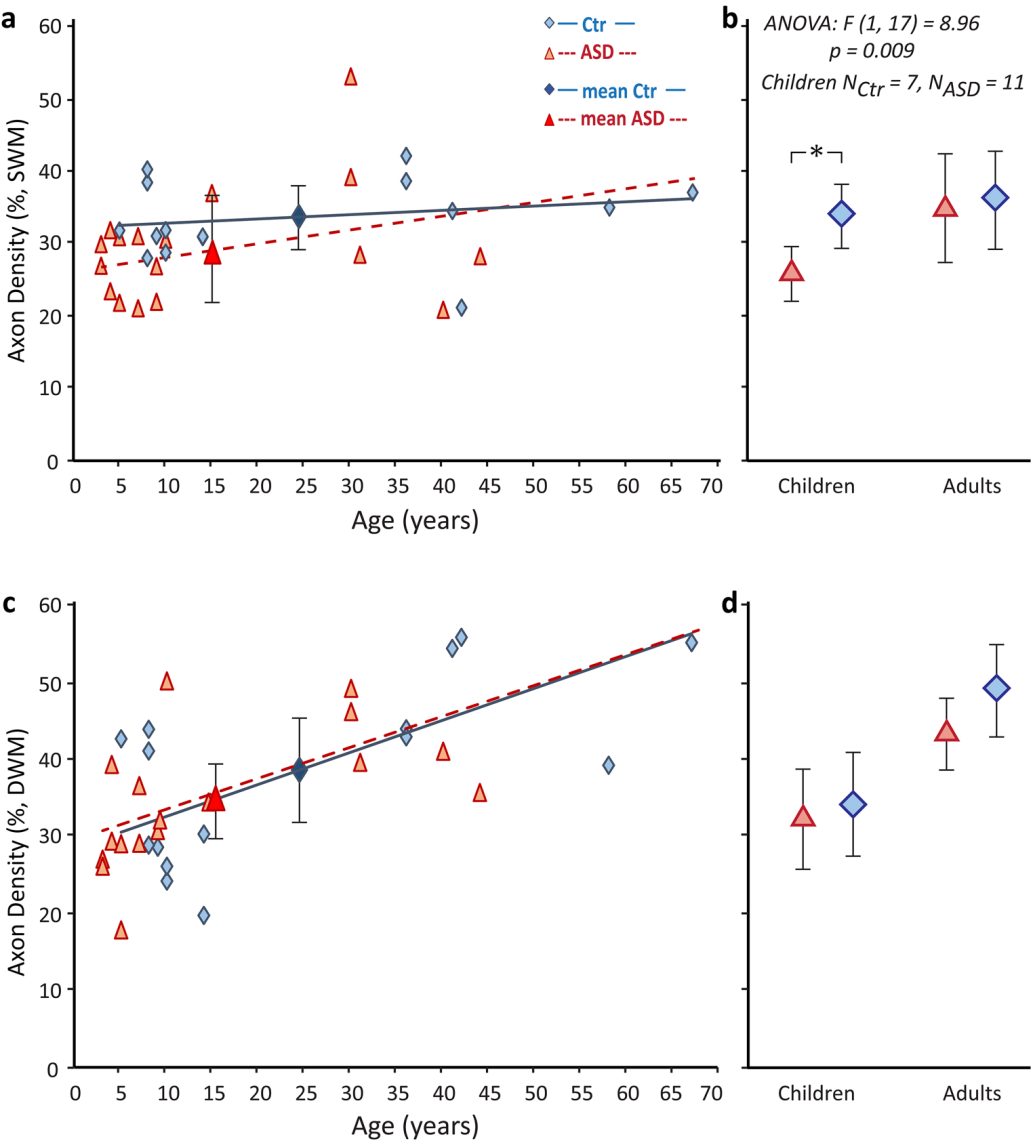
Postnatal development of axon density below ACC and changes in children with ASD. Estimated myelinated axon density in SWM **(a**, **b)** and DWM **(c**, **d)** below ACC, in children, adolescents and adults with and without ASD. Linear trend lines highlight upward trend for myelinated axon density with age in DWM. In the SWM (**a**, **b**), children with ASD had, on average, significantly lower density of myelinated axons compared to CTR children (* in **b** indicates significant difference, ANOVA, *p* = 0.009; mean ± SD for ASD and CTR children and adults). Fewer and overall thinner axons that travel in variable directions in the SWM, especially of children with ASD, are indicative of increased axon branching, in line with the high preponderance of pockets of paranodal cytoplasm in SWM axons (P in Fig. 1h), found in the vicinity of unmyelinated nodes at branching points. **a**, **c** Red triangles and red dotted line, ASD cases (large red triangle with error bars shows ASD mean ± SD). Blue diamonds and blue line, CTR cases (large blue diamond with error bars shows CTR mean ± SD). **b**, **d** Red triangles with error bars show ASD mean ± SD in children and adults, whereas blue diamonds with error bars show CTR mean ± SD

**Fig. 5:**
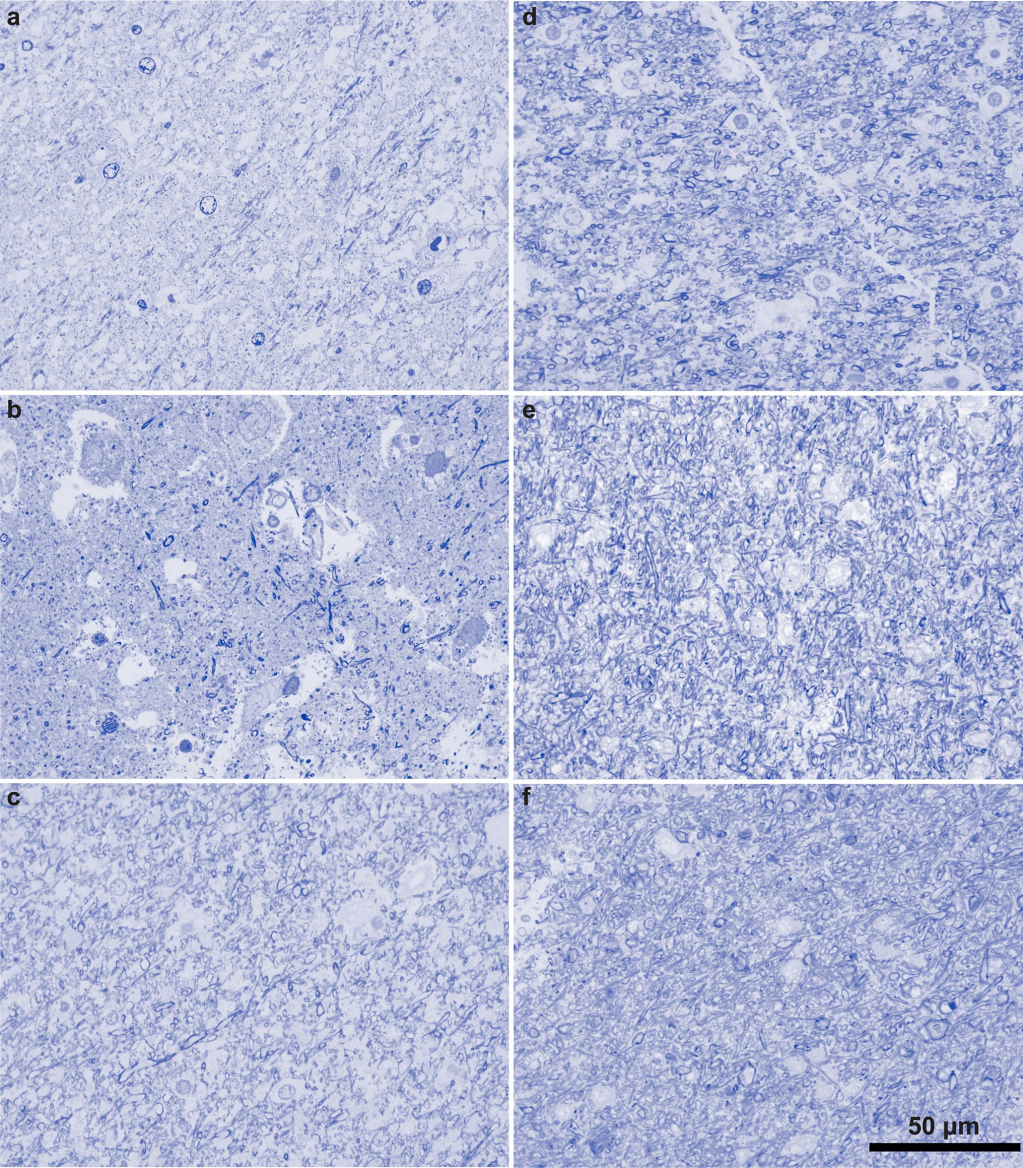
The superficial white matter below ACC in children with ASD had lower density of myelinated axons compared to CTR children. **a**–**f** Photomicrographs of 1-μm-thick coronal sections from the SWM below ACC stained with toluidine blue. **a**–**c** ASD cases, AN-08873 (**a**); AN-03221 (**b**); and 5308 (**c**). **d**–**f** CTR cases, 4337 (**d**); 451 (**e**); and 356 (**f**). Scale bar in f applies to all panels

**Fig. 6:**
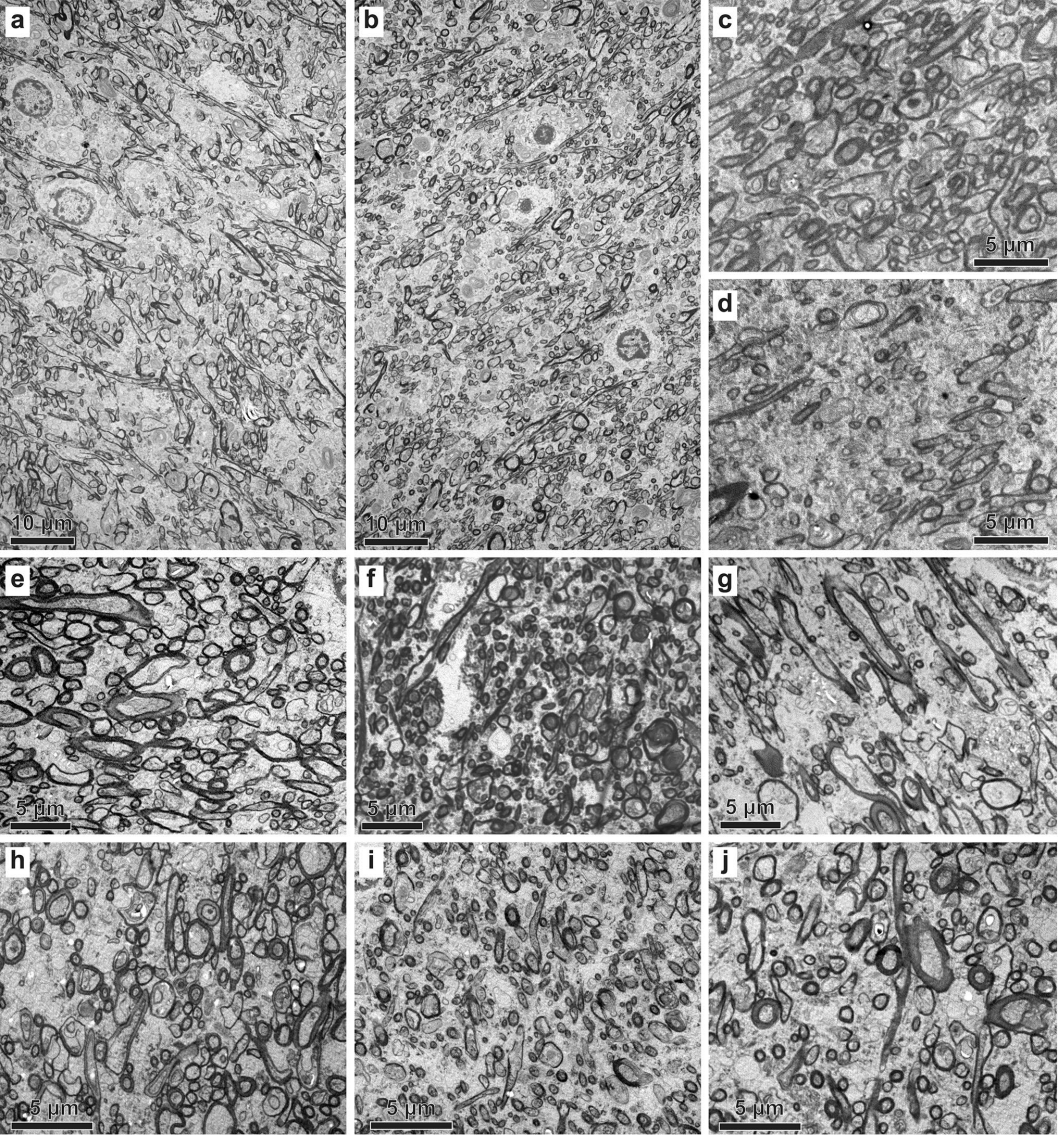
Representative EM photomicrographs of the white matter below ACC. (**a**, **d**, **h**, **i**, **j**) White matter in ASD cases 5308, AN-01293, AN-11989, AN-06746, and AN-07770, respectively. **b**, **c**, **e**, **f**, **g** White matter in CTR cases 4337, 451, B-6004, B-4786, and HAW, respectively. Approximately, 250,000 myelinated axons were analyzed at the EM level. Several oligodendrocytes are visible in panels **a**, **b**

**Fig. 7:**
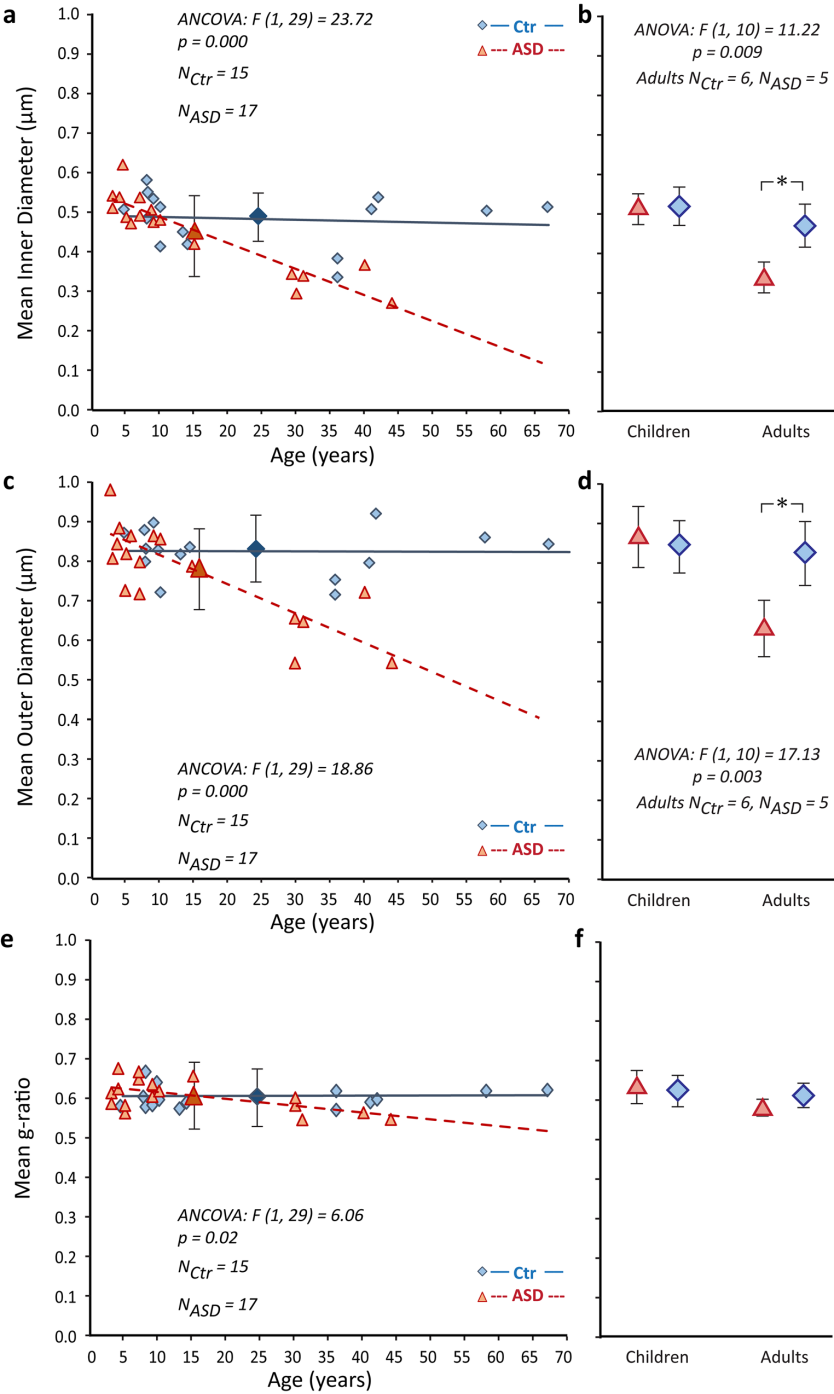
Altered developmental trajectory of axon size and conduction efficiency below ACC in ASD. **a**–**d** Development of myelinated axon size with age (inner diameter of axon profiles in **a**, **b** and outer diameter in **c**, **d**) in the white matter (SWM and DWM combined). Axon size below ACC did not change significantly across the lifespan of CTR individuals. Children with and without ASD initially had similar populations and thickness in myelinated axons, but in the course of development axons in the ASD group became significantly thinner in adulthood, producing a downward slope in the developmental trajectory of axon size (ANCOVA showed significantly different trajectories). Further comparison in children and in adults separately (**b**, **d**) highlighted the significant differences of the ASD and CTR groups in adulthood (ANOVA, *p* = 0.009 in **b**; and *p* = 0.003 in d; mean ± SD for ASD and CTR children and adults). **e**, **f** Postnatal development of the g-ratio (inner/outer axon diameter), which did not change during typical development but significantly decreased with age in ASD (ANCOVA in** e**). This decrease was due to the steeper decrease of the inner versus the outer axon diameter, suggesting thinning of the axolemma and not the myelin in ASD. The g-ratio is a reliable indicator of the efficiency of conduction velocity, with average values around 0.6 considered to be optimal for the efficient transmission of neural signals. In all panels: ASD cases, red triangles and red dotted lines (large red triangles with error bars in **a**, **c**, **e** show ASD mean ± SD); CTR cases, blue diamonds and blue lines, (large blue diamonds with error bars in **a**, ** c**,** e** show CTR mean ± SD). The slopes of the trend lines are significantly different between CTR and ASD groups in **a**, **c** and **e** (ANCOVA with age as a covariate). Panels **b**, **d**, and **f** show mean ± SD for ASD and CTR children and adults, and * in **b**, **d** indicate significant differences revealed with ANOVA

To tease apart changes in axon features with age, we studied their postnatal development in groups of children, adults and a small group of adolescents. Changes in size and density of myelinated axons below ACC were small with age. Neurotypical adults (36–67 years) had relatively more thick myelinated axons than children (4–10 years), who had a higher proportion of thin axons (Fig. 3). The density of myelinated axons increased with age in CTR and ASD groups, a trend that was more pronounced in the DWM (Fig. 4). Further analysis of the groups of children and adults separately revealed a significant difference between CTR and ASD cases. Children with ASD (3-10 years) had significantly lower density of myelinated axons in the SWM below ACC than the CTR group (Figs. 4a, b, 5, 6), suggesting an early pathology in this region. Axon size and density in both CTR and ASD teenagers (14–15 years) were well within the range seen in adults and children (Figs. 4, 7).

### Axon diameters showed opposite developmental trajectories in CTR and ASD groups

Myelinated axons below ACC did not change in average thickness in the CTR group between 4 and 67 years (Fig. 7). In contrast, in the ASD group, the mean inner and outer diameter of myelinated axons was lower in adults than in children, following a steep downward trajectory in thickness through the course of development (Fig. 7a–d). Postnatal thinning of axons was more pronounced (steeper) for the inner diameter, compared to the outer axon diameter, leading to a significant decrease in the g-ratio with age in ASD (Fig. 7e, f).

### Thin and thick axon populations showed opposite developmental trajectories in CTR and ASD groups

**Fig. 8:**
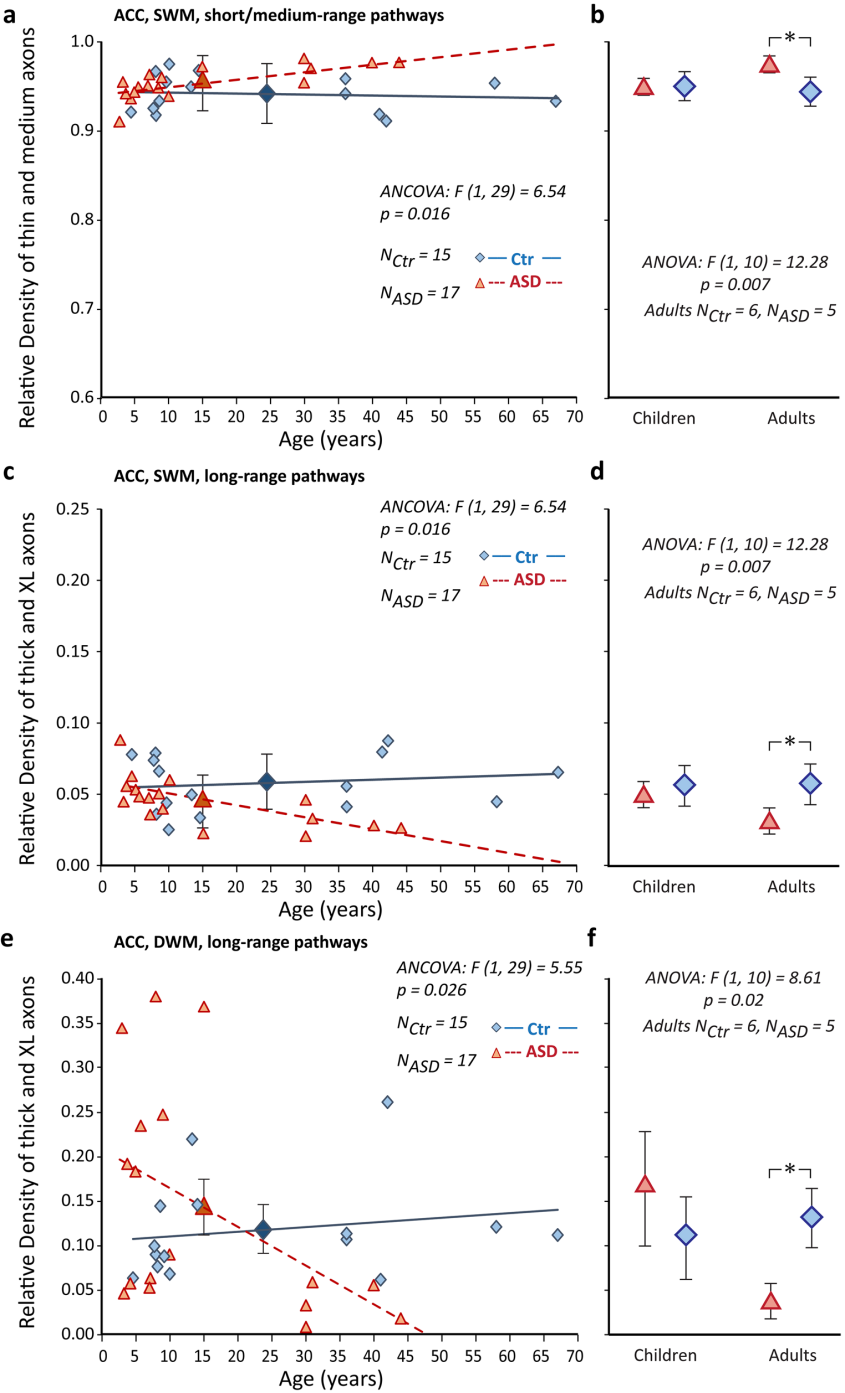
Altered developmental trajectories of short- and long-range pathways below ACC in ASD. **a**, **c**, **e** Postnatal developmental trajectory of short-/medium-range pathways in SWM (**a**), and long-range pathways in SWM (**c**) and DWM (**e**) below ACC that contain mostly thin and medium, or thick axons, respectively. In the CTR group, short- and medium-range pathways developed early and their relative proportion did not change with age. The relative proportion of long-range pathways showed a small, likely incremental trend for increase with age. ACC pathways in ASD showed significantly different developmental trajectories: short- and medium-range pathways increased in relative proportion with age, due to increase in the relative density of thin and medium axons. In contrast, the relative proportion of long-range pathways showed a significant decrease with age, based on the drop in the relative density of thick axons. **b**, **d**, **f** Further comparison in children and in adults separately highlighted the significant differences of the ASD and CTR groups in adulthood (ANOVA, *p* = 0.007 in **b** and **d**; and *p* = 0.02 in **f**; panels show mean ± SD for ASD and CTR children and adults). In all panels: ASD cases, red triangles and red dotted lines (large red triangles with error bars in **a**, **c**, **e** show ASD mean ± SD); CTR cases, blue diamonds and blue lines, (large blue diamonds with error bars in **a**, **c**, **e** show CTR mean ± SD). The slopes of the trend lines are significantly different between CTR and ASD groups in **a**, **c** and **e** (ANCOVA with age as a covariate). Panels **b**, **d**, and **f** show mean ± SD for ASD and CTR children and adults, and * in **b**, **d**, and **f** indicate significant differences revealed with ANOVA

The proportion of thin and medium axons present in childhood and adulthood was roughly constant in the CTR group, but increased with age in the ASD group, yielding more thin and medium axons (Fig. 8a, b). The slopes of the postnatal developmental trajectories between CTR and ASD groups differed significantly, reflecting the steep increase in the ratio of thin and medium axons with age in ASD, especially pronounced in the SWM (Fig. 8a). In contrast, the relative proportion of thick axons significantly declined with age in ASD, but followed an ascending trajectory in the CTR group, both in the SWM and DWM (Fig. 8c–f).

### Independent cross-validation of findings and cell counts in ACC gray matter

**Fig. 9:**
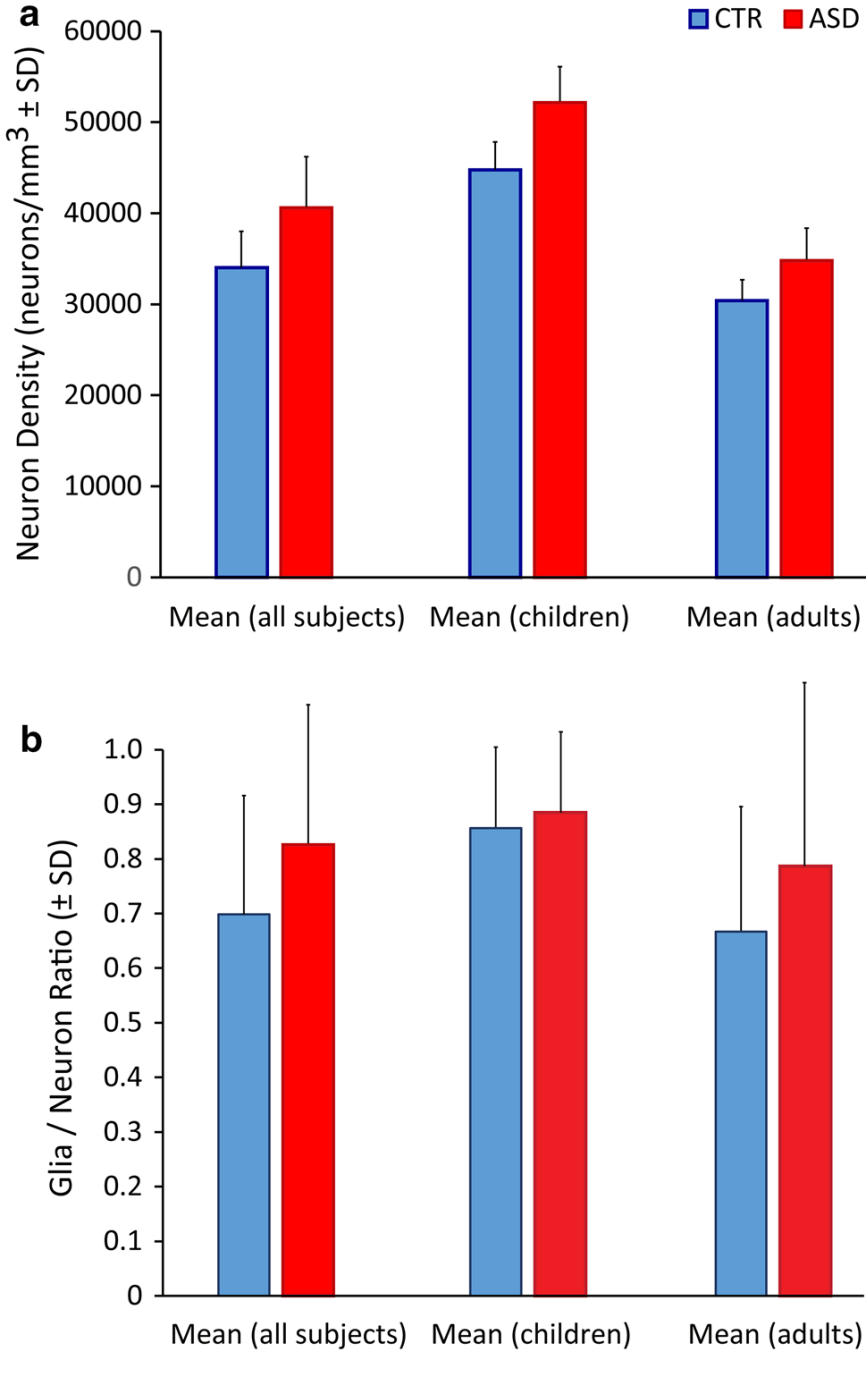
Cell density in ACC. **a** Neuron density ± standard deviation (SD) in CTR (left blue bar in each pair) and ASD (right red bar in each pair) groups showed no differences between the groups. **b** There were no differences in the glia to neuron ratio ± SD between CTR (left blue bar in each pair) and ASD (right red bar in each pair) groups. We included astrocytes and oligodendrocytes, but excluded microglia from the glia counts, because the latter are highly variable and depend on number and duration of pathological conditions and primary as well as secondary causes of death

We used two independent methods of optical and electron microscopy to assess the developmental trajectory of myelinated axons and changes across CTR and ASD groups, which yielded similar results. We investigated whether the changes in axons were accompanied by changes in the overlying neuronal density in the gray matter. We estimated cell densities in the ACC gray matter overlying the white matter analyzed, to examine whether changes in myelinated axon density or relative proportions of thin and thick axons are correlated with changes in the density of neurons and oligodendrocytes. The rationale was that lower density of axons may be due to lower density of neurons or fewer oligodendrocytes that myelinate axons. In contrast to axon changes, neuronal and glial density did not differ in the ACC gray matter across cases (Fig. 9), when estimated using a morphological feature algorithm we developed [28]. Estimation of neuron density in CTR and ASD groups showed no differences between groups. Interestingly, children had overall higher density of neurons in ACC compared to adults, in line with previous reports in cortical areas [11, 63, 64, 67, 84], and classical studies, which have shown that as the neuropil expands in postnatal development, the distance between neurons increases and therefore the density decreases in adulthood [13–17, 57]. In addition, there were no differences in the glia to neuron ratio between groups. For these estimates we included astrocytes and oligodendrocytes, but excluded microglia from the glia counts, because the latter are highly variable and depend on several factors, including the number and duration of pathological conditions and primary as well as secondary causes of death.

Finally, to further assess the robustness and generalizability of the findings we performed additional correlation analysis of several parameters that could confound the results. This analysis showed no potential effects of sex, PMI, medication, or other diagnoses (i.e., seizures) on all estimates for axon density, size, as well as cell densities at the 95% confidence level.

## Discussion

We provide direct evidence for typical and pathological changes in ACC white matter pathways with age. These findings were based on the largest cohort of human brains (32) and number of axons investigated (~ 1 million) at high resolution to date. Our findings revealed significant heterogeneity in ACC pathways in the course of postnatal development in ASD, in stark contrast to modest, possibly incremental, but biologically important, changes seen in typical postnatal development. Axon myelination in ACC pathways followed a heterogeneous trajectory, consistent with cortical changes seen with age in early childhood, adolescence and late adulthood (e.g., [7, 51, 58, 77]). Despite the heterogeneity, axon features were confined within ranges that were similar across cases. Consequently, we could detect small trends that reflect important processes during typical maturation of white matter pathways, as well as significant differences in the developmental trajectories of ACC pathways between CTR and ASD groups. In addition, our findings suggest that typical and pathological changes in the white matter below ACC during development may be gradual, supported by preliminary analyses of a few adolescent cases (*N* = 3). Further studies of the brain in adolescence will be required to reliably describe the rate of changes in the white matter from childhood to adulthood.

In typical postnatal development, the ratio of thick to thin axons and the density of myelinated axons increased from childhood to adulthood, consistent with the developmental processes of axon growth and myelination [25, 35, 51, 77]. The overall diameter of axons and their relative position within the white matter are indicators of their termination in nearby or distant brain areas [32, 62, 80]. Thus, thin and medium size axons, which were proportionally more abundant in the SWM (near cortical layer 6) link nearby areas. Our findings suggest that thin axons in SWM develop early during childhood and remain relatively unchanged into adulthood. On the other hand, thick axons, which are more prevalent in DWM and extend over long distances, develop later and increase with age, as shown here.

The detailed analyses of normal development of axons provided a foundation to compare with a pathologic state, which revealed significant differences in the density and thickness of myelinated axons below ACC in ASD. A key difference was the lower density of myelinated axons in the SWM below ACC in children with ASD, coinciding with the period when the white matter is enlarged [18, 32, 58]. This suggests that short-range pathways in the SWM that develop early, are disrupted early, and continue on an abnormal trajectory into adulthood, as reported previously for adults with ASD [80, 84]. Decreased axon density and increased spacing between myelinated axons, suggest that the early enlargement of the white matter in ASD [32, 58], could be due to an increase of unmyelinated axons. This hypothesis is supported by the lack of significant changes in the density of the overlying neurons or glia in ASD, even as the frontal microarchitecture changes by widening of minicolumns [12, 45] and ectopic patches of neurons [6, 61, 67, 74]. Increase in axon branching and proportion of unmyelinated axons in ASD may be due to high expression of the axon growth protein, GAP43 [3, 68, 80], which is also antagonistic to expression of basic myelin proteins [40]. These developmental features likely provide a milieu conducive to increased axon branching, thinning of axons in adulthood, and bias for reverberating short-range pathways from ACC to frontal areas. In contrast, the steep decrease in the relative proportion of thick axons that travel over long distances, may severely compromise long distance communication in ASD.

The opposite developmental trajectories of ACC pathways in CTR and ASD groups are also supported by the significant decrease with age in the thickness of myelinated axons in the ASD group, which remains relatively flat in typical development. The primary driver for the pathology in ASD is likely the thinning of the axolemma, but not the surrounding myelin, as evidenced by a steeper slope for the inner, compared to outer axon diameter in ASD. This change led to small but significant decrease in the g-ratio with age, a relationship that affects conduction velocity and neural synchrony between connected areas, in processes that are disrupted in ASD [52, 59].

Our study also provides an estimate of white matter axon trajectory anisotropy at the level of single axons, based on high-resolution microscopic imaging, and can be used to compare with measures obtained from diffusion tensor imaging (DTI) in living subjects, albeit at lower resolution. Axon parameters of density, thickness, and trajectory correlate well with imaging studies, and provide specific structural underpinnings of typical and pathological changes in the directional preference of diffusion [fractional anisotropy (FA)], and other estimated diffusion rates (molecular, axial, radial), which are associated with white matter integrity and myelination [2, 44, 65]. Comparison with imaging and histologic studies should provide reliable cross-validation of findings obtained with different scales of resolution to compare typical myelination and maturation of white matter cortical pathways in the human brain [25, 51, 71, 77]. Our approach for study of individual myelinated axons at high resolution can be scaled up for the systematic study of white matter pathways in health and various other pathological states [84].

In conclusion, we identified distinct changes from childhood to adulthood in the relative density, size composition, and trajectory of myelinated axons in the white matter below ACC. These novel findings revealed the developmental course in CTR brains and provided the basis to disentangle typical developmental processes from continued pathology in axons in ASD. Our findings revealed differences in the development of axons in ASD within a long lifespan, exhibiting a course that deviated from the CTR groups, biasing communication within the ACC and neighboring prefrontal cortices, and compromising communication over long distances.

## Electronic supplementary material

Below is the link to the electronic supplementary material.

Supplementary material 1 (PDF 20 kb)
