# Supplementary material for: Opposite development of short- and long-range anterior cingulate pathways in autism
Source: Acta Neuropathol. 2018 Sep 6;136(5):759–78. doi: 10.1007/s00401-018-1904-1 (PMC6208731; doi:10.1007/s00401-018-1904-1)
Supplement: Supplementary file 1 — Supplementary material 1 (PDF 20 kb) [file 401_2018_1904_MOESM1_ESM.pdf]

**SUPPLEMENTARY TABLE 1**

Clinical characteristics of subjects with autism

| **Subject number (ASD group)** | **Score on Autism Diagnostic Interview-Revised** | | | |
|---|---|---|---|---|
| | **Social Cutoff: 10** | **Communication Cutoff (V): 8 Cutoff (NV): 7** | **Restrictive and Repetitive Cutoff: 3** | **Early Abnormal Development Cutoff: 1** |
| 5144 | 28 | 20 (V), 12 (NV) | 3 | 3 |
| 4021 | 22 | 14 (NV) | 8 | 4 |
| 4029 | 20 | 8 (NV) | 3 | 3 |
| 5308[#] | 17 | 14 (V), 14 (NV) | 4 | 3 |
| 1182 | *** | *** | *** | *** |
| AN01293 (B-6349) | 26 | 12 (NV) | 5 | 4 |
| AN03221 | 27 | 16 (V) | 8 | 5 |
| AN03345 (B-6399) | 14 | 9 (NV) | 6 | 5 |
| AN13872 (B-7002) | ** | ** | ** | ** |
| AN08873 [##] (B-5569) | 22 | 14 (NV) | 6 | 5 |

| | | | | |
|---|---|---|---|---|
| AN04682 (B-7079) | 22 | 13 (V) | 5 | 5 |
| HSB4640[c] | *** | *** | *** | *** |
| AN-06746[a] (B-4541) | 26 | 18 (V), 13 (NV) | 6 | 5 |
| AN-18892[b] (B-4871) | 18 | 14 (V) | 6 | 3 |
| AN-08792[c] (B-5173) | 22 | 12 (NV) | 2* | 5 |
| AN-07770 (B-6232) | 12 | 14 (V) | 8 | 5 |
| AN-11989 (B-6677) | 26 | 22 (V) | 12 | 5 |

Other diagnosed disorders included, [a]: schizophrenia; [b]: depression; [c]: seizures.

[#]: Neuropathologic examination of 5308 suggested possible hypercellular cortex with microdysgenesis, partially confirmed by the stereologic neuron density counts we conducted. However, axon density and size were not affected.

[##]: Medical history and interviews indicate that this individual did not feel pain.

(V): verbal communication score; (NV): non-verbal communication score

*: score was below cutoff threshold due to physical limitations and poor motor skills of donor. However, family members reported repetitive behaviors at a younger age. With this exception, which is not an unusual pattern in the behavioral domain, all donors had difficulties with

communication, social behaviors, and atypical interests, consistent with a diagnosis of autism, and the ADI-R scores met and exceeded cutoffs for autism in each of these areas.

**: Moderately autistic based on Childhood Autism Rating Scale, Autism Diagnostic Observation Schedule (ADOS), and ADI. Moderately autistic range: 34.5, communication: 6 (autism cutoff 4); reciprocal social interactions 7 (autism cutoff 7).

***: Medical history indicates clinical ASD diagnoses, later confirmed by administration of ADI-R.
